# Priority-Aware Actuation Update Scheme in Heterogeneous Industrial Networks

**DOI:** 10.3390/s24020357

**Published:** 2024-01-07

**Authors:** Yeunwoong Kyung, Jihoon Sung, Haneul Ko, Taewon Song, Youngjun Kim

**Affiliations:** 1 Division of Information & Communication Engineering, Kongju National University, Cheonan-daero, Cheonan 31080, Republic of Korea; ywkyung@kongju.ac.kr; 2 Department of Electrical Engineering, KAIST, 291 Daehak-ro, Yuseong-gu, Daejeon 34141, Republic of Korea; sung.jh@kaist.ac.kr; 3 Department of Electronic Engineering, Kyung Hee University, Yongin-si 17104, Republic of Korea; heko@khu.ac.kr; 4 Department of Internet of Things, SCH MediaLabs, Soonchunhyang University, 22 Soonchunhyang-ro, Shinchang-myeon, Asan-si 31538, Republic of Korea; 5 School of Computer Science and Engineering, Kyungnam University, Changwon-si 51767, Republic of Korea

**Keywords:** actuation update, age of information, industrial networks, wireless networked control systems, Markov decision process, Q-learning

## Abstract

In heterogeneous wireless networked control systems (WNCSs), the age of information (AoI) of the actuation update and actuation update cost are important performance metrics. To reduce the monetary cost, the control system can wait for the availability of a WiFi network for the actuator and then conduct the update using a WiFi network in an opportunistic manner, but this leads to an increased AoI of the actuation update. In addition, since there are different AoI requirements according to the control priorities (i.e., robustness of AoI of the actuation update), these need to be considered when delivering the actuation update. To jointly consider the monetary cost and AoI with priority, this paper proposes a priority-aware actuation update scheme (PAUS) where the control system decides whether to deliver or delay the actuation update to the actuator. For the optimal decision, we formulate a Markov decision process model and derive the optimal policy based on Q-learning, which aims to maximize the average reward that implies the balance between the monetary cost and AoI with priority. Simulation results demonstrate that the PAUS outperforms the comparison schemes in terms of the average reward under various settings.

## 1. Introduction

According to Industry 4.0, wireless networked control systems (WNCSs) have been applied to industrial networks for various services, such as industrial automation, smart manufacturing, and unmanned robot control [1,2]. WNCSs have been considered as a prominent solution in industrial networks to provide real-time and reliable actuation [3,4]. Compared to conventional NCSs based on wired networks, since WNCSs are spatially distributed wireless control systems, they have been researched with respect to the enhancement of energy-harvesting capabilities [5], wireless resource scheduling [6], energy-aware performance optimization [7], and wireless attacks [8]. WNCSs generally consist of sensors, actuators, and a controller. Sensors collect the latest samples of environmental states and deliver them to the controller. After the controller computes control decisions for actuators, it sends the control command to the actuators. In addition, as mobile actuators, such as mobile robots and automated guided vehicles, have recently been deployed, wireless control for mobile actuators has been actively deployed. During the general process of WNCSs, there are two principal updates: (1) status updates from sensors to the controller, and (2) actuation updates from the controller to actuators, which need timely updates due to the time-critical control applications in WNCSs.

Since timeliness is an important metric in WNCSs, the age of information (AoI) has been introduced as a novel metric to quantify the freshness of information updates [9,10]. AoI is defined as the amount of elapsed time since the latest delivered information (i.e., updates in WNCSs) was generated. It is based on the perspective of destinations, and therefore it linearly increases with time until an update is received at a destination. Specifically, an update that was generated at time *u* has AoI *t* − *u* at a time *t* ( t≥u ). The update is said to be fresh when its AoI is close to zero. Since WNCSs require timely and fresh updates to improve their control performance, AoI has been applied to WNCSs as a key performance metric [11,12].

After AoI was introduced, the research on it for status updates in industrial networks or WNCSs has been maturely studied [2,12,13]. However, research on AoI for actuation updates has not been a focus, even though it is critical for control performance. For example, delayed actuation updates can result in production inefficiency, plant destruction, and casualties [2,13]. In other words, the timeliness of the actuation update should be controlled by the controller in the WNCS. In addition, since the impact of AoI on the actuation update can be different according to the control priorities for the actuators (i.e., robustness of AoI of the actuation update) [14,15], the priority needs to be considered when delivering the actuation update. For example, priority can be defined to classify purposes concerning the criticality or safety level at a particular moment [15]. Accordingly, the actuation updates of high priority are more sensitive to the changes in AoI than those of low priority. Consequently, priorities should be incorporated with AoI.

Meanwhile, in industrial environments, heterogeneous wireless networks, such as cellular (e.g., 5G new radio (NR)) and WiFi networks [16,17], have been deployed. Accordingly, the type of network available for mobile actuators varies depending on the location. In this scenario, the actuation updates via cellular networks engender a monetary cost while the updates via WiFi networks are usually free to use. To reduce the monetary cost, the control system prefers to use WiFi networks for the actuation updates. Note that, compared to cellular networks that offer perfect coverage (i.e., always available), WiFi networks are distributed and therefore can be exploited in an opportunistic manner (i.e., they are intermittently available) [18]. This means that when the control system needs to deliver the actuation update, it can use the cellular network immediately. However, to reduce the monetary cost, the control system can deliver the update via a WiFi network after waiting until a WiFi network has become available, which can increase AoI. As explained above, the increased AoI results in a critical situation, especially for high-priority control commands. Consequently, it is important to determine the appropriate actuation update policy, considering both the monetary cost and AoI with priority.

To address the AoI control problem considering heterogeneous networks, there have been several works [18,19,20,21,22,23]. These can be categorized into the following: (1) status update design [19,20,21,22]; and (2) actuation update design [18,23]. Pan et al. [19] determined the scheduling policy to transmit status updates over an unreliable but fast channel or a slow, reliable channel to minimize AoI. A Markov decision process (MDP) model was exploited to formulate and solve the optimal scheduling problem. Bhati et al. [20] analyzed the average AoI with heterogeneous multiple servers and determined the optimal routing parameter between the servers to minimize the average AoI. For the system model, M/M/1 queuing models with different service rates among the servers were assumed. Fidler et al. [21] showed the effect of independent parallel channels on AoI based on the queuing models. Specifically, G/G/1 queuing models with Markov channels were used for the parallel systems with a time-varying capacity. Xie et al. [22] formulated the generalized scheduling problem in multi-sensor multi-server systems to minimize AoI. This paper jointly considered link scheduling, server selection, and service preemption, and formulated an MDP problem to find the optimal policy. As explained above, these papers addressed the AoI control problem considering heterogeneous networks. However, these papers mainly focused on status updates (i.e., irrespective of the actuation update) and did not consider priority. Altman et al. [18] and Raiss-el-fenni et al. [23] introduced the receiver’s policy to decide whether to receive updates from cellular or WiFi networks to minimize costs. They focused on the receiver’s perspective about whether to activate the device or not. However, it is difficult for the specific application to control device activation without the user’s involvement. Consequently, it is more suitable to determine the policy from the control system (i.e., application server), as proposed in this paper. In addition, these papers did not consider the priority as well.

To address these challenges, this paper proposes a priority-aware actuation update scheme (PAUS) that jointly considers cost and AoI with priority. In the PAUS, the control system determines whether to deliver or delay the actuation update to the actuator based on AoI with priority and cost. We formulate a Markov decision process (MDP) model and determine the optimal policy based on Q-learning (QL). Simulation results demonstrate that the PAUS reduces the cost while satisfying the required AoI.

The main contributions of this paper are as follows: (1) to the best of our knowledge, this is the first work where the actuation update is determined jointly considering AoI and monetary cost; (2) the increasing rate of AoI is determined according to the control priorities (i.e., robustness of AoI of the actuation update) to consider the different impact of AoI with priority on the actuation update; (3) an MDP model is formulated to maximize the average reward that implies the balance between AoI with priority and monetary cost; (4) the optimal policy on whether to deliver or delay the actuation update to the actuator can be obtained using QL; and (5) extensive simulation results present the performance of the PAUS under various settings, which can be utilized as the guidelines for the control system operator.

The remainder of this paper is organized as follows. The system model and problem formulation are provided in Section 2 and Section 3, respectively. The QL-based algorithm is presented in Section 4. After simulation results are provided in Section 5, this paper is concluded with future works suggested in Section 6.

## 2. System Model

**Figure 1:**
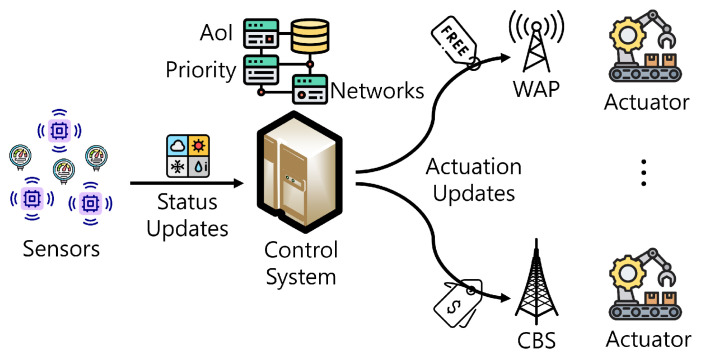
System model.

Figure 1 presents the system model of this paper. In our model, a control system (i.e., the controller) delivers the actuation update to the mobile actuator using either the cellular base station (CBS) or a WiFi access point (WAP). As we mentioned above, the CBS is always available, whereas a WAP is only available when the actuator is close enough to the WAP [18]. In addition, since the control system (i.e., the controller) delivers the update, we assume that the transmission energy can be ignored. Moreover, it is assumed that there is no transmission error because the model is focused on the actuation update delivery (i.e., it is not focused on physical communication) [24,25].

The monetary cost should be considered for actuation updates according to the network type. The use of the cellular network (i.e., via the CBS) requires monetary costs based on the data plans of network operators. On the other hand, the use of WiFi networks (i.e., via a WAP) is usually free. Therefore, the actuation update via a WAP is efficient in terms of reducing the monetary cost for the system operator. However, WAPs are intermittently available [18]. Consequently, actuation updates using WAPs in an opportunistic manner can lead to increased AoI while reducing the monetary cost. Since increasing AoI leads to a critical situation (e.g., production inefficiency and casualties [2,13]), it is necessary to maintain low AoI. Moreover, since there are control priorities in actuation updates, the priority should be considered when delivering the actuation updates. For example, an update of a high priority has a stricter AoI requirement than that of a low priority, which is not relatively sensitive to AoI [15]. Note that the AoI requirement is set including the transmission time between the controller and actuator.

**Figure 2:**
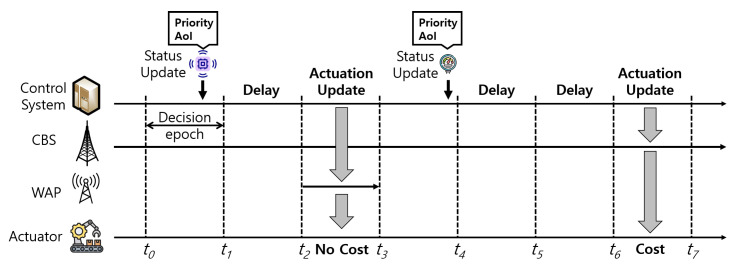
Timing diagram for PAUS.

Figure 2 shows the specific timing diagram for the PAUS. At each decision epoch (e.g., $t_{0}$ , $t_{1}$ , …, $t_{7}$ ), the control system determines whether to deliver or delay the actuation update, considering the available network of the actuator (i.e., CBS or WAP), the current AoI, and the priority of the update. In Figure 2, the thick horizontal solid lines from the CBS and WAP denote the availability of the network. For example, a WAP is available between $t_{2}$ and $t_{3}$ , while CBS is always available. When the control system receives a status update, it can wait for a WAP to reduce the monetary cost. If a WAP becomes available, the control system delivers the update via the WAP (e.g., between $t_{2}$ and $t_{3}$ ) without monetary cost. Otherwise, the control system should deliver the update via the CBS (e.g., between $t_{6}$ and $t_{7}$ ) before exceeding the target AoI requirement, even though the update engenders monetary costs.

Therefore, it is important to determine an actuation update policy that can minimize monetary cost while maintaining AoI below a desired value, considering priority. To determine the optimal policy, this paper formulates an MDP problem in the next section.

## 3. Problem Formulation

In this section, we formulate an MDP model based on the timing diagram in Figure 2. In the formulated MDP model, the actuation update can be delivered via either the CBS or a WAP. Furthermore, if a WAP is not currently available, the control system can delay the update with the expectation of future WAP contact.

Whether to deliver the actuation update (i.e., via the CBS or a WAP) or delay the update is determined at each decision epoch $t\in T=\left\{1,2,3\ldots \right\}$ according to the state at the decision epoch.

### 3.1. State Space

At each decision epoch, the state set S can be defined as

(1) $$\begin{array}{c} S=L\times \prod_{i}V_{i}\times \prod_{i}E_{i} \end{array}$$

where **L** denotes the availability of WAPs. In addition, $V_{i}$ and $E_{i}$ represent the current AoI and the existence of the actuation update with priority *i*, respectively.

First, L can be defined as

(2) $$\begin{array}{c} L=\left\{0,1\right\} \end{array}$$

where l(∈L) represents whether the actuator can receive the information from a WAP or not. In other words, l=0 means that the actuator cannot connect to a WAP (i.e., can only connect to the CBS) because there is no available WAP. Otherwise (i.e., l=1 ), the actuator can connect to a WAP as well as the CBS because a WAP is close to the actuator.

Moreover, $V_{i}$ can be defined as

(3) $$\begin{array}{c} V_{i}=\left\{0,\ldots ,V_{m}\right\} \end{array}$$

where $v_{i}\;\left(\in V_{i}\right)$ denotes the current AoI of the actuation update with priority *i* and $V_{m}$ is the maximum AoI in the system model.

In addition, $E_{i}$ can be defined as

(4) $$\begin{array}{c} E_{i}=\left\{0,1\right\} \end{array}$$

where $e_{i}\;\left(\in E_{i}\right)$ denotes the existence of the actuation update with priority *i*. In other words, $e_{i}=1$ means that the actuation update with priority *i* exists at the control system and needs to be delivered to the actuator. Otherwise (i.e., $e_{i}=0$ ), the actuation update with priority *i* does not exist.

### 3.2. Action Space

At each decision epoch, the control system determines an action (i.e., deliver or delay). Consequently, let $A=\prod_{i}A_{i}$ denote a global action space for the actuator, where $A_{i}$ is a local action space of the actuation update with priority *i*. $A_{i}$ can be defined as

(5) $$\begin{array}{c} A_{i}=\left\{0,1\right\} \end{array}$$

where 0 and 1 stand for defined actions. Specifically, $a_{i}\;\left(\in A_{i}\right)=0$ means that the control system delivers the update to the actuator. On the other hand, $a_{i}=1$ means that the control system delays the update.

### 3.3. Transition Probability

The transition probability from the current state s(∈S) to the next state $s^{\prime }\left(\in S\right)$ when the control system chooses the action *a* can be described as

(6) $$\begin{array}{c} P\left[s^{\prime }|s,a\right]=P\left[l^{\prime }|l\right]\times \prod_{i}P\left[v_{i}^{\prime },e_{i}^{\prime }|v_{i},e_{i},a_{i}\right], \end{array}$$

because the availability of a WAP is not dependent on the other states or determined action. In addition, the existence of the update is not dependent on the other states while the current AoI is dependent on the existence of the update. Consequently, $P[v_{i}^{\prime },e_{i}^{\prime }|v_{i},e_{i},a_{i}]$ can be rearranged as

(7) $$\begin{array}{c} P\left[v_{i}^{\prime },e_{i}^{\prime }|v_{i},e_{i},a_{i}\right]=P\left[v_{i}^{\prime }|v_{i},e_{i},a_{i}\right]\times P\left[e_{i}^{\prime }|e_{i},a_{i}\right]. \end{array}$$

We assume that the duration of the disconnection (connection) between a WAP and an actuator follows the exponential distribution with mean $1/\lambda^{D}$ ( $1/\lambda^{C}$ ) [24,25]. Consequently, the probability that the actuator can connect to a WAP during τ is $\lambda^{C}\tau$ . In addition, the actuator can disconnect with a WAP during τ with probability $\lambda^{D}\tau$ . Therefore, $P[l^{\prime }|l=0]$ and $P[l^{\prime }|l=1]$ can be defined by

(8) $$\begin{array}{c} P\left[l^{\prime }|l=0\right]=\left\{\begin{array}{c} 1-\lambda^{C}\tau ,\;\mathrm{if}\;l^{\prime }=0\\ \lambda^{C}\tau ,\;\;\;\;\;\;\mathrm{if}\;l^{\prime }=1 \end{array}\right. \end{array}$$

and

(9) $$\begin{array}{c} P\left[l^{\prime }|l=1\right]=\left\{\begin{array}{c} 1-\lambda^{D}\tau ,\;\mathrm{if}\;l^{\prime }=1\\ \lambda^{D}\tau ,\;\;\;\;\;\;\mathrm{if}\;l^{\prime }=0. \end{array}\right. \end{array}$$

If the control system delays the actuation update when the update exists, the current AoI increases until $V_{m}$ . If $v_{i}$ becomes $V_{m}$ , the control system should deliver the actuation update to the actuator. In this paper, AoI increases with different increasing rates according to the priority *i*. This is because, even if the same amount of time elapses, it can be perceived as relatively more time for the update with a high priority (i.e., higher *i*) compared to that with a low priority (i.e., lower *i*). In other words, the increasing rate of high-priority updates (e.g., high criticality level) is higher than that of low-priority updates (e.g., low criticality level) because an increasing AoI of high-priority updates is much more critical compared to that of low-priority updates. Moreover, when the control system delivers the actuation update, the corresponding AoI becomes 0. Consequently, $P[v_{i}^{\prime }|v_{i},e_{i},a_{i}]$ can be described as

(10) $$\begin{array}{c} P\left[v_{i}^{\prime }|0\leq v_{i}<V_{m},e_{i}=1,a_{i}=1\right]=\left\{\begin{array}{c} 1,\;\;\mathrm{if}\;v_{i}^{\prime }=v_{i}+h\left(i\right)\\ 0,\;\;\mathrm{otherwise}, \end{array}\right. \end{array}$$

(11) $$\begin{array}{c} P\left[v_{i}^{\prime }|v_{i}=V_{m},e_{i}=1\right]=\left\{\begin{array}{c} 1,\;\;\mathrm{if}\;v_{i}^{\prime }=0\\ 0,\;\;\mathrm{otherwise}, \end{array}\right. \end{array}$$

(12) $$\begin{array}{c} P\left[v_{i}^{\prime }|v_{i},e_{i}=0\right]=\left\{\begin{array}{c} 1,\;\;\mathrm{if}\;v_{i}^{\prime }=0\\ 0,\;\;\mathrm{otherwise}, \end{array}\right. \end{array}$$

and

(13) $$\begin{array}{c} P\left[v_{i}^{\prime }|v_{i},e_{i}=1,a_{i}=0\right]=\left\{\begin{array}{c} 1,\;\;\mathrm{if}\;v_{i}^{\prime }=0\\ 0,\;\;\mathrm{otherwise} \end{array}\right. \end{array}$$

where h(i) is an increasing function (e.g., a linear increasing function) as the priority *i* increases.

We assume that the probability that a new actuation update with priority *i* occurs following a Poisson distribution with mean $\lambda_{i}^{U}$ [26]. Consequently, the probability that the control system has a new actuation update with priority *i* during τ is $\lambda_{i}^{U}\tau$ . Therefore, $P[e_{i}^{\prime }|e_{i},a_{i}]$ can be described as

(14) $$\begin{array}{c} P\left[e_{i}^{\prime }|e_{i},a_{i}=0\right]=\left\{\begin{array}{c} \lambda_{i}^{U}\tau ,\;\;\;\;\;\;\;\mathrm{if}\;e_{i}^{\prime }=1\\ 1-\lambda_{i}^{U}\tau ,\;\;\mathrm{if}\;e_{i}^{\prime }=0, \end{array}\right. \end{array}$$

(15) $$\begin{array}{c} P\left[e_{i}^{\prime }|e_{i}=0,a_{i}=1\right]=\left\{\begin{array}{c} \lambda_{i}^{U}\tau ,\;\;\;\;\;\;\;\mathrm{if}\;e_{i}^{\prime }=1\\ 1-\lambda_{i}^{U}\tau ,\;\;\mathrm{if}\;e_{i}^{\prime }=0, \end{array}\right. \end{array}$$

and

(16) $$\begin{array}{c} P\left[e_{i}^{\prime }|e_{i}=1,a_{i}=1\right]=\left\{\begin{array}{c} 1,\;\;\mathrm{if}\;e_{i}^{\prime }=1\\ 0,\;\;\mathrm{if}\;e_{i}^{\prime }=0. \end{array}\right. \end{array}$$

### 3.4. Reward and Cost Functions

For the reward and cost functions, we consider the monetary and delivery costs as well as the current AoI. Specifically, the total reward function, r(s,a) , is defined as

(17) $$\begin{array}{c} r(s,a)=wg(s,a)-(1-w)f(s,a) \end{array}$$

where g(s,a) is the reward function by means of AoI and f(s,a) is the cost function according to the monetary and delivery cost. Note that the delivery cost denotes the additional cost caused by the delivery, such as energy consumption or association overhead [18]. *w* ( 0≤w≤1 ) is a weight factor to balance g(s,a) and f(s,a) .

Specifically, g(s,a) can be obtained by

(18) $$g\left(s,a\right)=\sum_{i}\left(-{\left\{\delta_{cur,i}\left(t\right)-\tau_{target,i}\right\}}_{+}\right),$$

where $\delta_{cur,i}\left(t\right)$ is the current AoI with priority *i* at the current time *t* and $\tau_{target,i}$ is the target AoI, which can be considered as a service requirement of the update with priority *i*. In addition, $x_{+}$ means the ramp function, defined as

(19) $$\begin{array}{c} x_{+}=\left\{\begin{array}{cc} x, & \mathrm{if}\;x\geq 0,\\ 0, & \mathrm{otherwise}. \end{array}\right. \end{array}$$

In addition, f(s,a) can be represented as

(20) $$\begin{array}{c} f\left(s,a\right)=\left\{\begin{array}{cc} C_{m}+C_{t}, & \mathrm{if}\;a=0,\\ 0, & \mathrm{otherwise} \end{array}\right. \end{array}$$

where $C_{m}$ and $C_{t}$ are the monetary and delivery costs when the control system delivers the actuation update. These $C_{m}$ and $C_{t}$ are predefined constants that allow the balancing of the monetary cost and the delivery cost within the cost function and thus define priorities.

## 4. QL-Based Actuation Update Algorithm

To find the optimal policy in the formulated MDP model in Section 3, this paper proposes a QL-based algorithm. QL is a typical reinforcement learning algorithm to solve sequential decision problems [27] with low computational complexity [27,28] and low memory usage [29]. QL uses a state-action value, Q(s,a) , with a given state *s* and an action *a*. After Q(s,a) is initialized to zero, Q(s,a) can be updated at each subsequent iteration by

(21) $$Q\left(s,a\right)⟵Q\left(s,a\right)+\alpha (R+\gamma \max_{a^{\prime }\in A}Q\left(s^{\prime },a^{\prime }\right)-Q\left(s,a\right))$$

where α , *R*, and γ denote the learning rate, instant reward, and discount factor, respectively. To balance between exploitation and exploration, the decaying ϵ -greedy approach can be used for iterative updates of Q(s,a) . Specifically, the agent (i.e., control system) randomly selects the action with probability ϵ and selects the greedy action with maximum Q(s,a) with probability 1 − ϵ . In addition, ϵ gradually decreases during iterative updates to initially explore the environment and to finally exploit the greedy action. After Q(s,a) converges to the optimal, the best action for every state can be selected as $\arg\max_{a}Q\left(s,a\right)$ . Detailed steps for the Q(s,a) update are given in Algorithm 1. As shown in Algorithm 1, if the convergence condition is satisfied (lines 9–10), we can obtain the optimal policy (line 11). Otherwise, Q(s,a) is iteratively updated (lines 2–8).

Note that because state and action spaces are not large in the current system model as defined in Section 2, QL is exploited to solve the formulated problem with low computational complexity [27,28] and low memory usage [29]. In our future work, when state and action spaces become larger than those in the current system model, deep reinforcement learning approaches, such as a deep deterministic policy gradient, will be considered, which have strong performance in handling larger state and action spaces [30,31].
**Algorithm 1** Steps for 
Q(s,a)
 update
  1:Initialize parameters: Q(s,a) ( s∈S , a∈A ), initial probability ϵ , count value *c*, learning rate α , discount factor γ , episode length *T*.  2:Copy current Q(s,a) to $Q_{c}\left(s,a\right)$ for comparison of changes after one episode.  3:**for** each episode from 1 to *T* **do**  4:   At each step of the decision epoch, observe the current state *s*  5:   Use ϵ -greedy approach to select an action *a*  6:   Calculate the reward *R* and observe the next state $s^{\prime }$
  7:   Update Q(s,a) according to (21)  8:**end for**  9:If every element in $|Q\left(s,a\right)-Q_{c}\left(s,a\right)|\leq \alpha$ , c⟵c+1 .10:If c=10 , go to the next step. Otherwise, ϵ⟵max(0.01,ϵ−0.001) and go to step 2.11:Compute optimal policy $\arg\max_{a}Q\left(s,a\right)$ .


## 5. Performance Analysis Results

To evaluate the performance, we conduct extensive simulations by means of a Python-based event-driven simulator, where each simulation includes 10,000 decision epochs, and the average values of 10 simulations are used for the average reward. We compare the proposed scheme (i.e., PAUS) with the following four schemes: (1) SEND, where the control system delivers the actuation update immediately when a new actuation update occurs to minimize AoI, (2) TARGET, where the control system delays the actuation update and then delivers it right before exceeding the target AoI requirement, (3) PERIOD, where the control system periodically delivers the actuation update, and (4) WAIT, where the control system waits for WiFi to make the best use of WiFi.

The default parameter settings are as follows. The average probability of disconnection and connection between the WAP and actuator are set to 0.4 and 0.2 [25], respectively. The default values of $V_{m}$ and *w* are set to 20 and 0.7 , respectively. In addition, h(i) is assumed to be a linear function with a static coefficient (i.e., 1) according to *i*. Furthermore, we assume that there are five priorities, where one is the lowest (i.e., less critical) and five is the highest (i.e., more critical) [15]. Moreover, $\tau_{target,i}$ , $\lambda_{i}^{U}$ , and the period of PERIOD are set to 10, 0.3 , and 10 decision epochs, respectively. It is assumed that $C_{m}$ and $C_{t}$ to use CBS are set to 4 and 1, respectively, while those to use WAP are set to 0 and 1, respectively. For the Q(s,a) update, we assume that α , γ , *T*, and ϵ are set to 0.2 , 0.95 , 1000, and 0.99 , respectively. Although default parameter settings are assumed, since these parameter settings can be different between scenarios, we will provide the effect of these parameters (i.e., changes in the weight factor, actuation update arrival rates, monetary cost, and WAP connection probability) on the performances in the following analysis.

**Figure 3:**
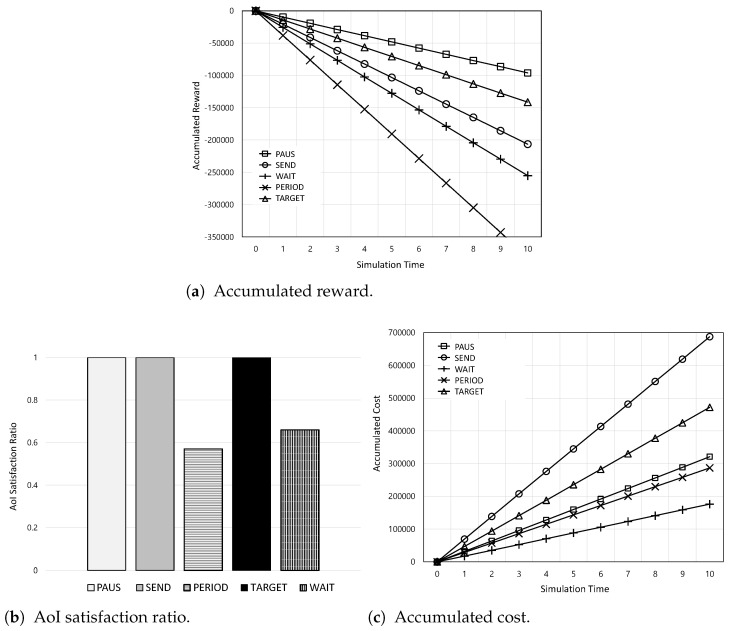
Overall performance of the accumulated reward, AoI satisfaction ratio, and accumulated cost according to the simulation time.

Figure 3 shows the overall performance of the accumulated reward, AoI satisfaction ratio, and total monetary cost according to the simulation time. In Figure 3a, as the simulation time increases, the accumulated rewards for all schemes decrease because AoI and the monetary cost are accumulated. Among them, the PAUS achieves the highest accumulated reward because it jointly considers AoI and monetary cost. On the other hand, WAIT has the lowest accumulated reward because it waits for WiFi, which leads to increased AoI. Meanwhile, in Figure 3b, it is found that the PAUS, SEND, and TARGET can guarantee the AoI requirement (i.e., 100% satisfaction ratio), while PERIOD and WAIT cannot. This is because PERIOD and WAIT deliver the actuation update periodically and only with WiFi, respectively, without consideration of AoI. In addition, Figure 3c shows the accumulated cost among them. Among the PAUS, SEND, and TARGET, which have 100% satisfaction ratios, it can be noted that the PAUS has the lowest accumulated cost. This means that the PAUS can minimize the monetary cost while maintaining AoI within the required value.

**Figure 4:**
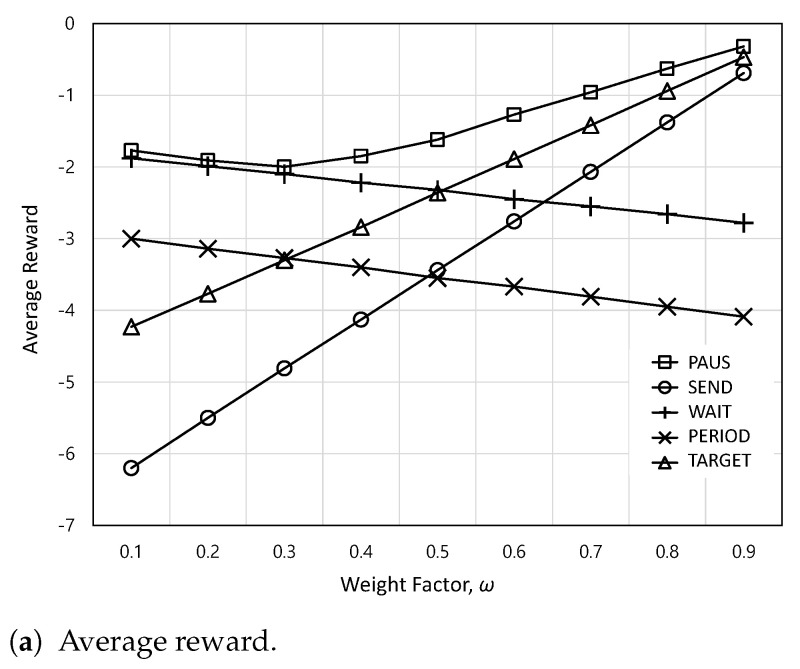

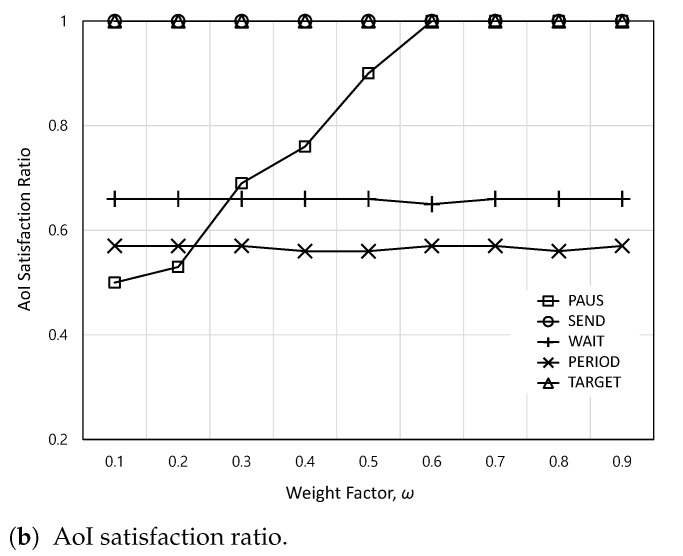
The average reward and AoI satisfaction ratio according to weight factor *w*.

Figure 4 shows the average reward and AoI satisfaction ratio according to weight factor *w*. In Figure 4a, as *w* increases, the average rewards of PERIOD and WAIT decrease because of the increasing AoI. Between them, the average reward of WAIT is higher than that of PERIOD because it tries to reduce AoI whenever WiFi is available. On the other hand, as *w* increases, the expected rewards of SEND and TARGET increase due to the reduced AoI. Between them, the increasing rate of SEND is higher than that of TARGET because SEND can minimize AoI with increasing *w*. Meanwhile, the PAUS achieves the highest average reward. This is because it can reduce monetary cost at a lower *w* and AoI at a higher *w*. In Figure 4b, the PAUS cannot guarantee the AoI requirement at a lower *w* compared to SEND and TARGET, which can satisfy the AoI requirement. This is because, at a lower *w*, the PAUS aims to reduce the monetary cost, which can increase AoI, to maximize the total reward function defined in (17). On the other hand, SEND and TARGET can achieve 100% AoI satisfaction ratios because SEND and TARGET try to deliver the actuation update immediately when a new update occurs and before exceeding the target AoI requirement, respectively. However, they need higher monetary costs, which finally reduce the average reward, as shown in Figure 4a. Consequently, for the PAUS, it is found that *w* needs to be set higher than 0.6 to guarantee the AoI requirement.

**Figure 5:**
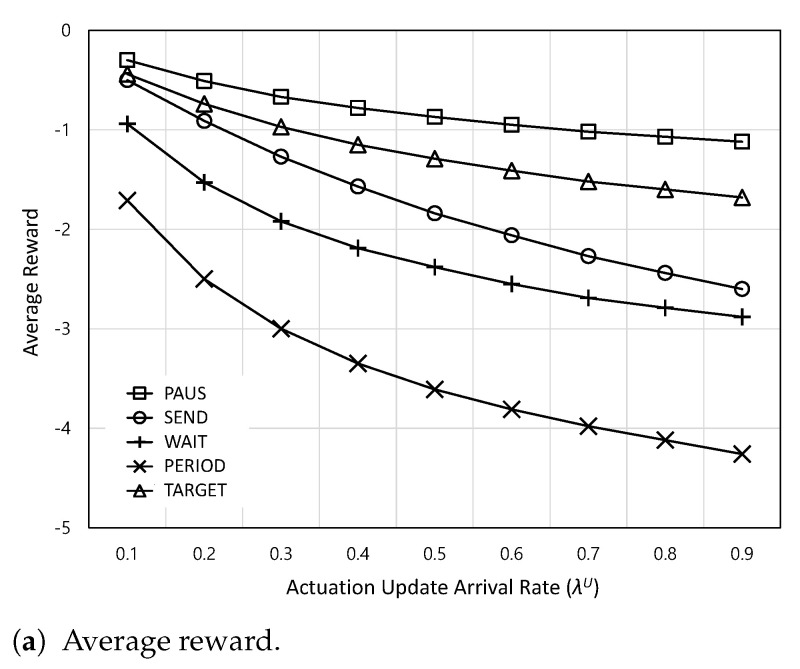

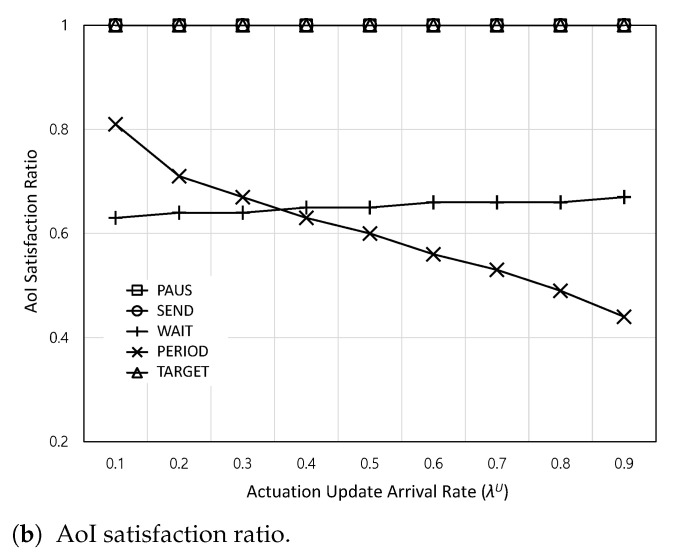
The average reward and AoI satisfaction ratio according to the actuation update arrival rate $\lambda^{U}$ .

Figure 5 shows the average reward and AoI satisfaction ratio according to the actuation update arrival rate $\lambda^{U}$ . In Figure 5a, as $\lambda^{U}$ increases, the average rewards of all schemes decrease because an increasing $\lambda^{U}$ increases the number of deliveries, which can lead to monetary costs or delayed updates. Among them, the decreasing rate of SEND and PERIOD is higher than that of others. In the case of SEND, this is because as $\lambda^{U}$ increases, the number of updates via the CBS becomes higher, which increases the monetary cost. On the other hand, in the case of PERIOD, the periodical actuation update is still used even when $\lambda^{U}$ increases, which results in delayed updates. Overall, the PAUS achieves the highest average reward because it aims to minimize the cost jointly considering the monetary cost and AoI. In addition, as shown in Figure 5b, even when $\lambda^{U}$ increases, the PAUS, SEND, and TARGET can guarantee the AoI requirement. On the other hand, PERIOD and WAIT cannot guarantee the AoI requirement because PERIOD still uses the periodical actuation update and WAIT delays the actuation update and waits for WiFi irrespective of $\lambda^{U}$ changes.

**Figure 6:**
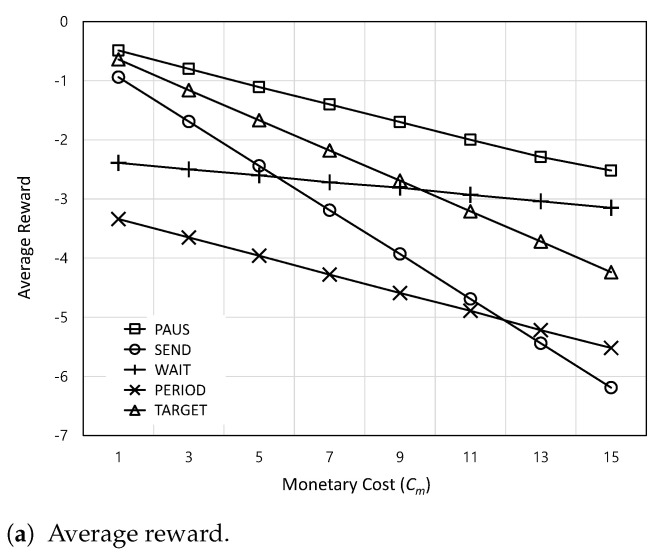

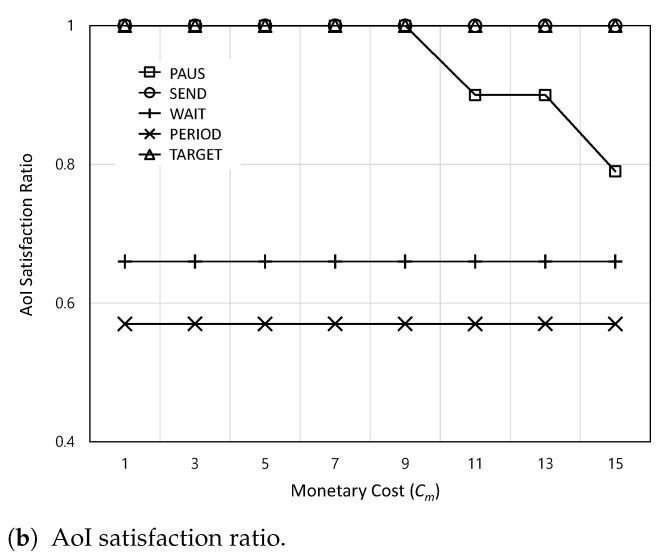
The average reward and AoI satisfaction ratio according to the monetary cost $C_{m}$ .

Figure 6 shows the average reward and AoI satisfaction ratio according to the monetary cost $C_{m}$ . In Figure 6a, as $C_{m}$ increases, the average rewards of all schemes decrease because increased $C_{m}$ leads to higher monetary cost. Among them, the decreasing rate of SEND is higher than that of others because SEND immediately tries to deliver the actuation update even when only the CBS is available. On the other hand, WAIT has the lowest decreasing rate because WAIT always prefers to use a WAP. Overall, the PAUS achieves the highest average reward. This is because the PAUS can fully utilize either the CBS at a lower $C_{m}$ or a WAP at a higher $C_{m}$ . In Figure 6b, it is shown that the PAUS cannot guarantee the AoI requirement at a higher $C_{m}$ compared to SEND and TARGET, which can satisfy the AoI requirement. This is because at a higher $C_{m}$ , the PAUS aims to reduce the monetary cost, which can increase AoI to maximize the total reward function defined in (17). On the other hand, SEND and TARGET try to deliver the actuation update without consideration of the monetary cost, which finally reduces the average reward, as shown in Figure 6a. Note that, if the system operator needs to enhance the AoI satisfaction ratio, even at a higher $C_{m}$ , the weight factor *w* in the total reward function can be adjusted.

**Figure 7:**
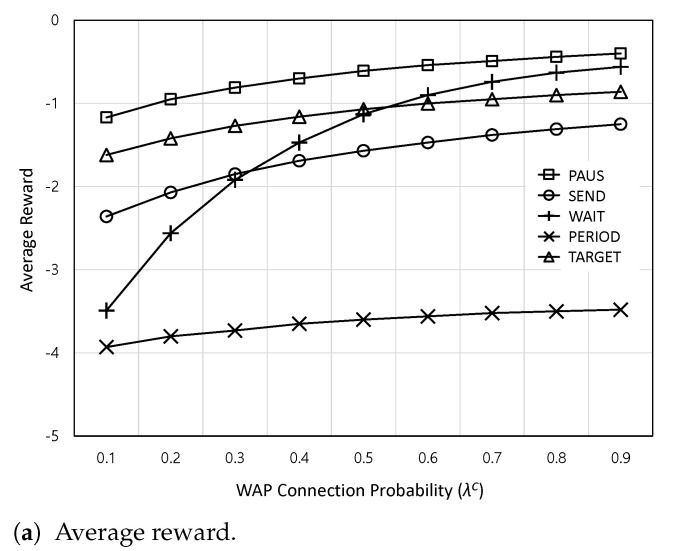

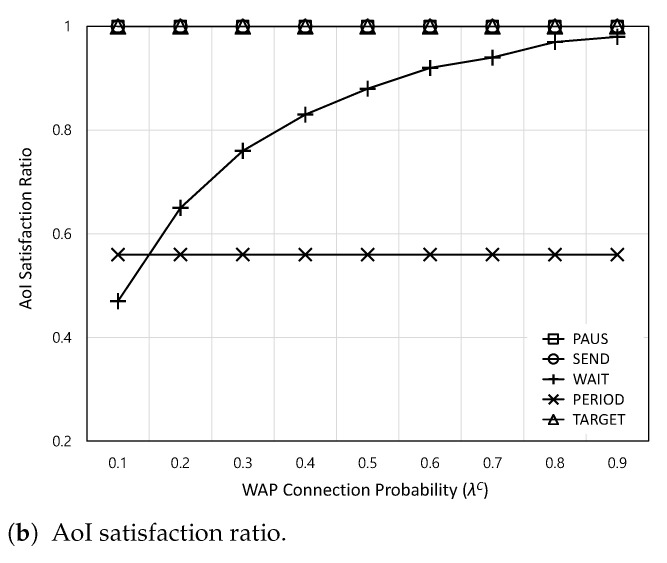
The average reward and AoI satisfaction ratio according to the WAP connection probability $\lambda^{C}$ .

Figure 7 shows the average reward and AoI satisfaction ratio according to the WAP connection probability $\lambda^{C}$ . In Figure 7a, as $\lambda^{C}$ increases, the expected rewards of all schemes increase because an increased $\lambda^{C}$ leads to lower monetary cost. Among them, the increasing rate of WAIT is higher than that of others because the increasing $\lambda^{C}$ results in more opportunities to deliver updates via a WAP, which can reduce AoI, as also shown in Figure 7b. Overall, as presented in Figure 7a,b, the PAUS achieves the highest average reward while guaranteeing the AoI requirement. This is because the PAUS can fully utilize either the CBS at a lower $\lambda^{C}$ or a WAP at a higher $\lambda^{C}$ .

**Figure 8:**
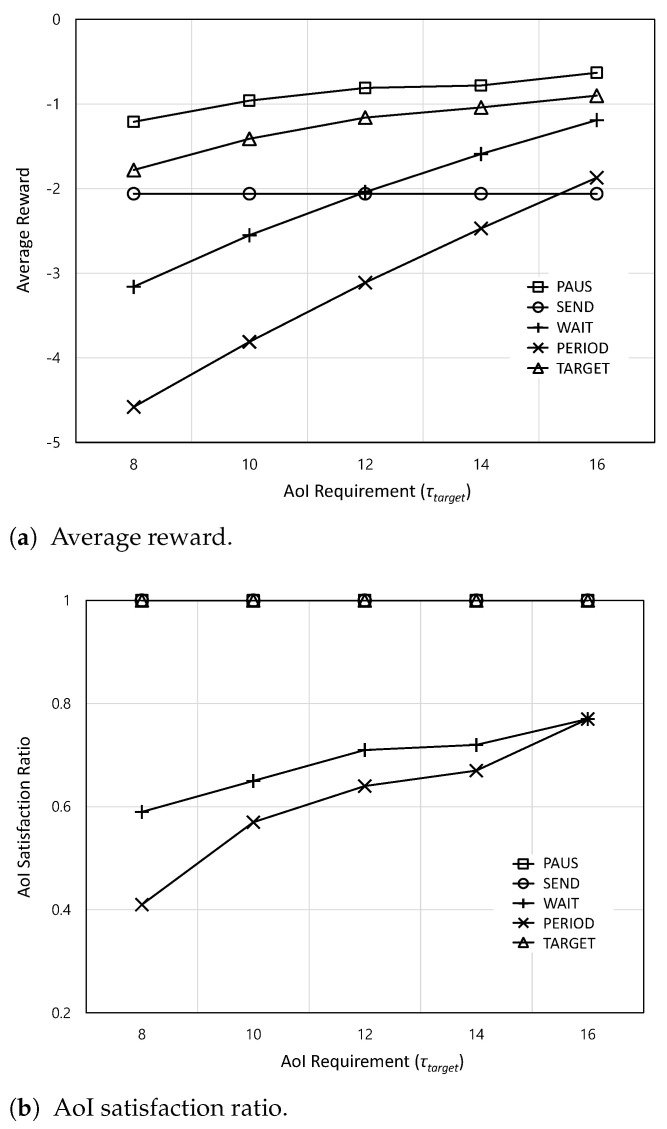
The average reward and AoI satisfaction ratio according to the AoI requirement $\tau_{target}$ .

Figure 8 shows the average reward and AoI satisfaction ratio according to the AoI requirement $\tau_{target}$ . In Figure 8a, as $\tau_{target}$ increases, the average rewards of all schemes except for SEND (i.e., PAUS, WAIT, PERIOD, and TARGET) increase because there is enough time to wait for WiFi, which can reduce the monetary cost. However, because SEND delivers actuation updates irrespective of the AoI requirement, the average reward of SEND does not change according to the AoI requirement. From Figure 8b, although the AoI satisfaction ratios of WAIT and PERIOD increase, they still cannot guarantee the AoI requirement.

## 6. Conclusions

This paper proposes a priority-aware actuation update scheme (PAUS), where the control system determines whether to deliver or delay the actuation update, considering monetary cost and AoI with priority. To find the optimal policy, this paper formulates an MDP model and provides a QL-based solution to maximize the average reward, which implies the balance between AoI with priority and monetary cost. The simulation results demonstrated that the PAUS outperforms the comparison schemes in terms of the expected reward. In addition, it is shown that the average reward is influenced by the weight factor, actuation update arrival rate, monetary cost, and WiFi access point connection probability. Moreover, it is also found that the PAUS can minimize the monetary cost while maintaining AoI below the required value by adjusting the weight factor. In our future work, the PAUS will be integrated into the non-public network (or private 5G) architecture to provide industrial network solutions, such as priority-based wireless control of mobile robots and automated guided vehicles.

## Data Availability

Data are contained within the article.
